# Supplementary material for: Remote ischemic conditioning counteracts the intestinal damage of necrotizing enterocolitis by improving intestinal microcirculation
Source: Nat Commun. 2020 Oct 2;11:4950. doi: 10.1038/s41467-020-18750-9 (PMC7532542; doi:10.1038/s41467-020-18750-9)
Supplement: Supplementary file 1 — Supplementary Information (Figures) [file 41467_2020_18750_MOESM1_ESM.docx]

**Supplementary Information**

**Remote ischemic conditioning counteracts the intestinal damage of necrotizing enterocolitis by improving intestinal microcirculation**

Koike, Li, Ganji, Zhu, et al.


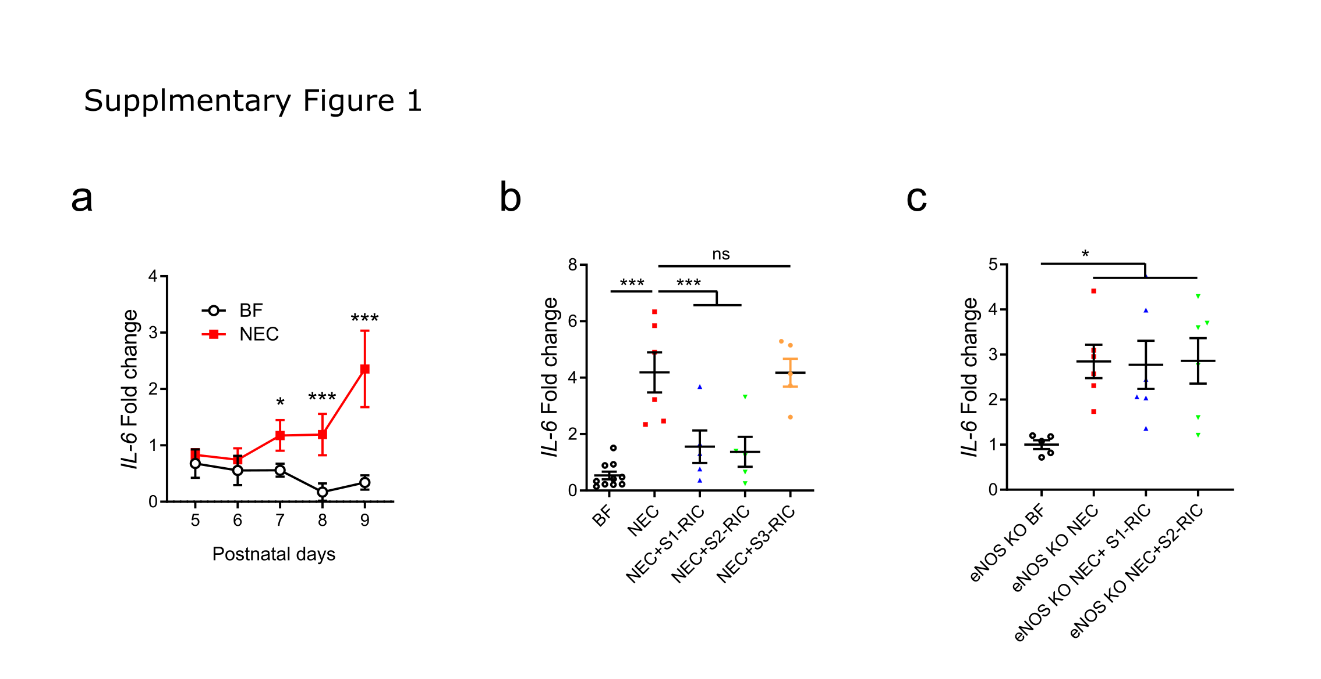


**Supplementary Figure 1.** Stage 1 and 2 RIC reduce intestinal inflammation in wildtype but not *eNOS* knockout mice. **a** To establish the timing of RIC regimen in the initial development of NEC, intestinal inflammation was investigated in C57BL/6 mouse pups from P5 to P9 (n=4 for BF P5, P6; n=5 for BF P7, P8; n=3 for BF P9; n=5 for NEC P5, P6, P9, n=4 for NEC P7, n=3 for NEC P8). **b** Effect of Stage 1, 2, and 3 RIC on intestinal inflammation in NEC pups at P9 (BF: n=10, NEC: n=6, NEC+Stage 1 RIC/Stage 2 RIC/Stage 3 RIC=5). **c** Effect of Stage 1 and 2 RIC on intestinal inflammation in *eNOS* knockout NEC pups (n=6 per group). mRNA expression levels of pro-inflammatory marker *IL-6* was measured with SYBR green-based qPCR. Data was compared using two-sided one-way ANOVA with post hoc Turkey test (*p<0.05; *p<0.01, ***p<0.001), and data are presented as mean ± SEM. Source data are provided as a Source Data file.


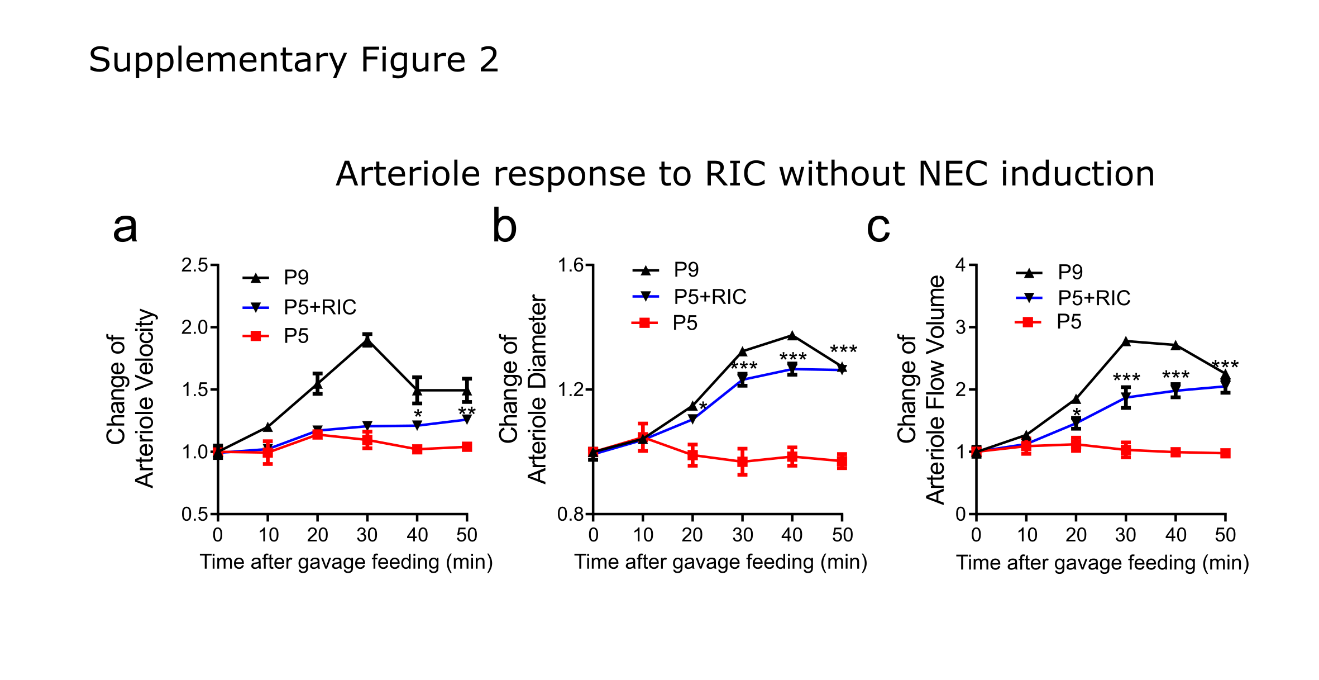


**Supplementary Figure 2.** RIC counteracts the poor intestinal hemodynamic response to single formula feeding in the early neonatal period, measured *in vivo* using TPLSM in real time. There was increased submucosal arteriole **a** velocity (µm/s), **b** diameter (µm), and **c** flow volume [(μm)^3^/s] after formula feeding in P9 but not P5 pups. Conditioning with a single episode of RIC prior to feeding counteracted the poor response to feeding in P5 pups showing increased submucosal arteriole velocity (µm/s), diameter (µm), and flow volume [(μm)^3^/s]. Data was compared using two-sided one-way ANOVA with post hoc Turkey test (n=3 per group; *p<0.05; *p<0.01, ***p<0.001). Data are presented as mean ± SEM. Source data are provided as a Source Data file.


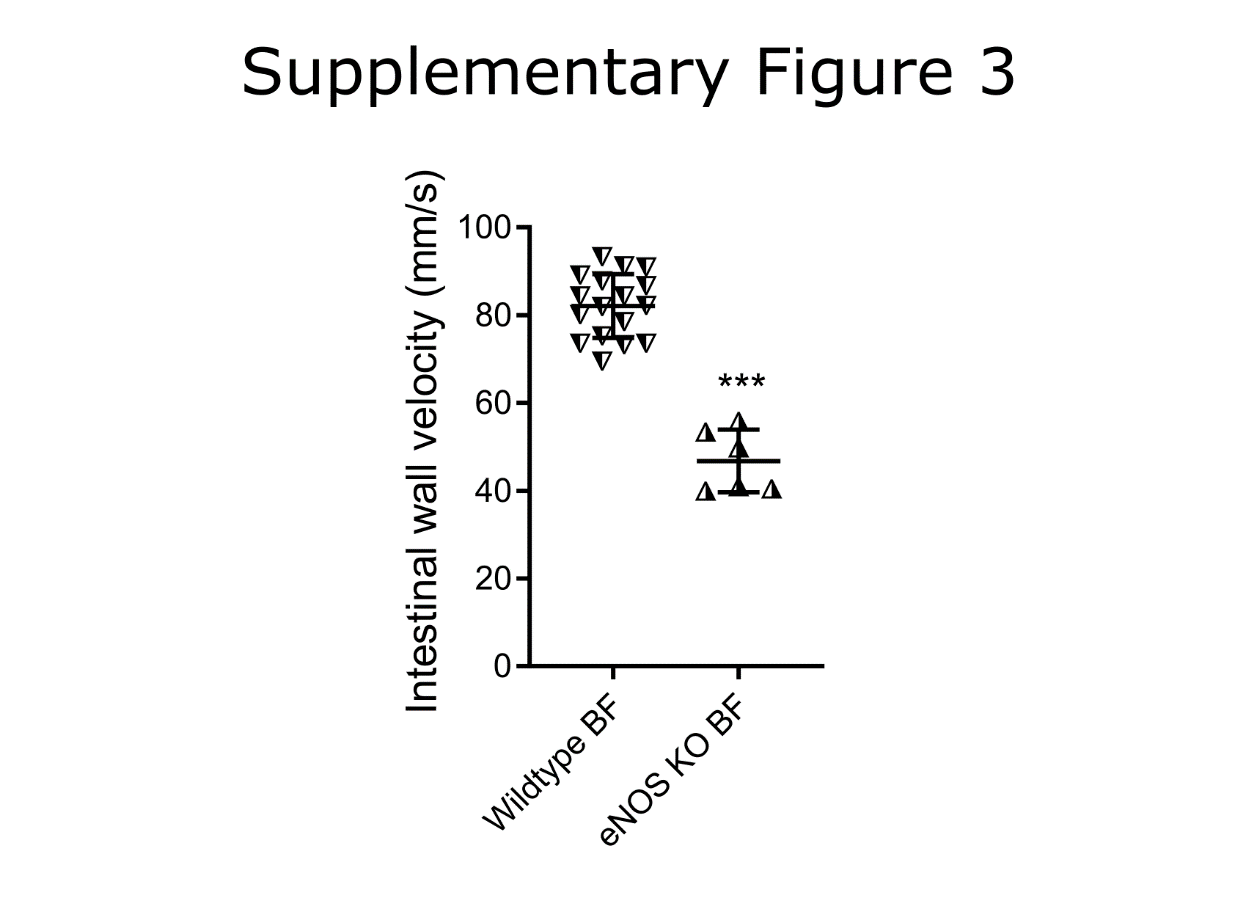


**Supplementary Figure 3.** Intestinal wall flow velocity (mm/s) in *eNOS* knockout breastfed control pups was measured with Doppler ultrasound showing 50% reduction in intestinal wall perfusion compared to wild type breastfed pups (BF: n=6; minimum of 2 readings obtained per pup; *eNOS* KO BF: n=6)**.** Data was compared using two-sided one-way ANOVA with post hoc Turkey test (*p<0.05; *p<0.01, ***p<0.001), and data are presented as mean ± SEM. Source data are provided as a Source Data file.


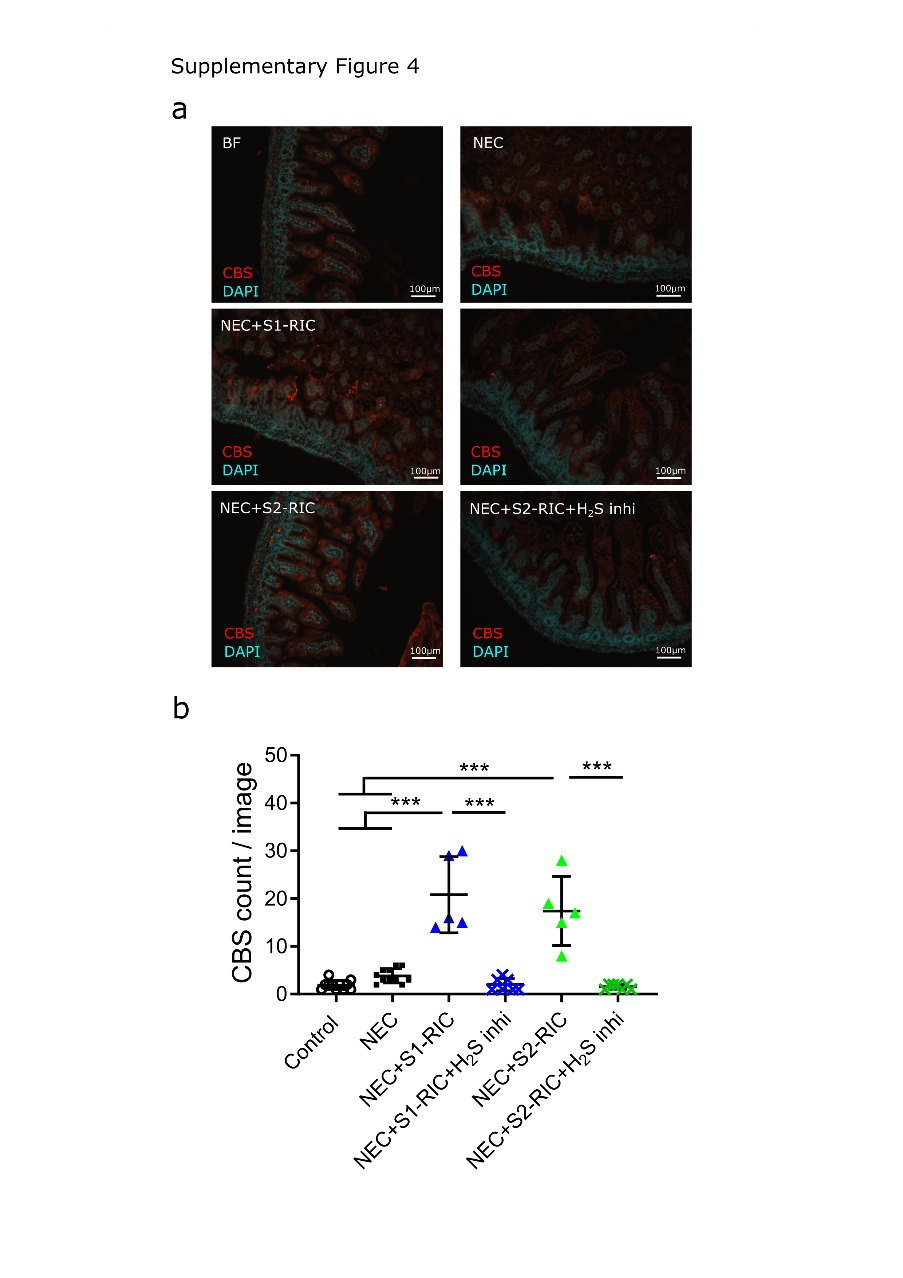


**Supplementary Figure 4.** Stage 1 and 2 RIC increased expression of H_2_S-synthesizing enzyme in the ileum during NEC, but not following treatment with H_2_S-synthesizing enzyme inhibitors. **a** Immunofluorescent localization of cystathionine-β-synthase (CBS) in the ileum of breastfed (BF) control (n=11), NEC (n=11), NEC with Stage 1 RIC (n=5) or Stage 2 RIC (n=5), and NEC with Stage 1 RIC (n=7) or Stage 2 RIC (n=7) treated with H_2_S-synthesizing enzyme inhibitors. CBS staining (red) was primarily localized to cells within the submucosa, and lamina propria. Scale bars are equivalent to 100 µm in the images shown. **b** Quantification of CBS expression in the ileum of pups in the listed experimental groups. Data was compared using two-sided one-way ANOVA with post hoc Turkey test (***p<0.001), and data are presented as mean ± SEM. Source data are provided as a Source Data file.


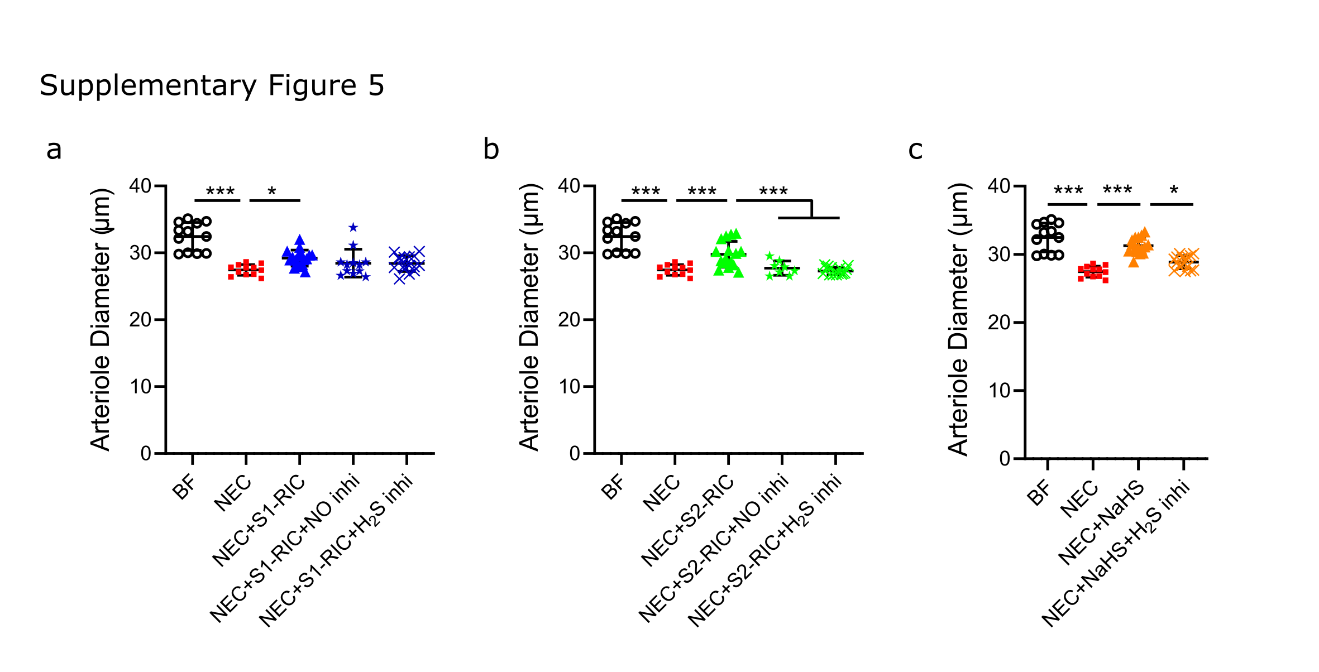


**Supplementary Figure 5.** Assessment of submucosal arteriole diameter (µm) measured *in vivo* using TPLSM movie analysis in real time. **a** Arteriole diameter in NEC pups receiving Stage 1 RIC did not change in response to treatment with NO-synthase inhibitor or H_2_S-synthesizing enzyme inhibitors. Arteriole diameter in NEC pups receiving Stage 2 RIC was lower in response to treatment with H_2_S-synthesizing enzyme inhibitors but did not change in response to NO-synthase inhibitor. Arteriole diameter in NEC pups receiving NaHS, exogenous H_2_S donor, was higher compared to NEC alone, but was decreased in response to treatment with H_2_S-synthesizing enzyme inhibitors. **b** Intestinal mRNA expression of *IL-6,* pro-inflammatory marker, was increased in NEC pups receiving Stage 1 or 2 RIC, following administration of NO-synthase inhibitor or H_2_S-synthesizing enzyme inhibitor. Treatment with NaHS reduced mRNA expression of *IL-6* in NEC pups, but not following administration of H_2_S-synthesizing enzyme inhibitors. mRNA expression levels of pro-inflammatory marker *IL-6* was measured with SYBR green-based qPCR. Data was compared using two-sided one-way ANOVA with post hoc Turkey test (n=5 per group; minimum of 2 readings were obtained per group; *p<0.05; **p<0.01; ***p<0.001), and data are presented as mean ± SEM. Source data are provided as a Source Data file.


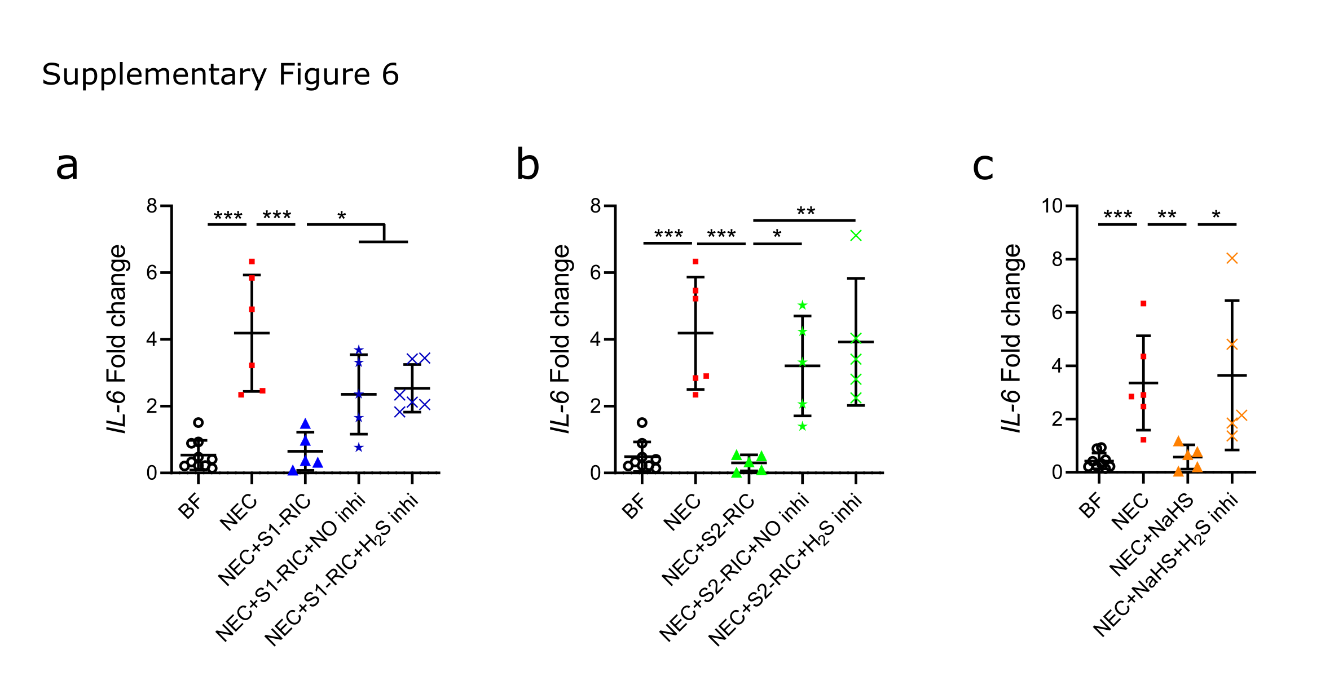


**Supplementary Figure 6.** RIC-mediated preservation of intestinal perfusion via nitric oxide and hydrogen sulfide is required to attenuate intestinal inflammation during NEC. mRNA expression of *IL-6,* measured using qRT-PCR, was elevated in NEC pups receiving **a** Stage 1 RIC, and **b** Stage 2 RIC, following administration of NO-synthase inhibitor or H_2_S-synthesizing enzyme inhibitors. **c** Treatment with NaHS attenuated intestinal inflammation in NEC pups, but not following treatment with H_2_S-synthesizing enzyme inhibitors. Data was compared using two-sided one-way ANOVA with post hoc Turkey test (BF: n=10; NEC: n=6; NEC+Stage 1 or 2 RIC: n=5; NEC+Stage 1 or 2 RIC+NO inhibitors: n=5; NEC+Stage 1 RIC+H2S inhibitors: n=6; NEC+Stage 2 RIC+H_2_S inhibitors: n=6; NEC+NaHS: n=5; NEC+NaHS+H_2_S inhibitors: n=5; *p<0.05; **p<0.01; ***p<0.001), and data are presented as mean ± SEM. Source data are provided as a Source Data file.


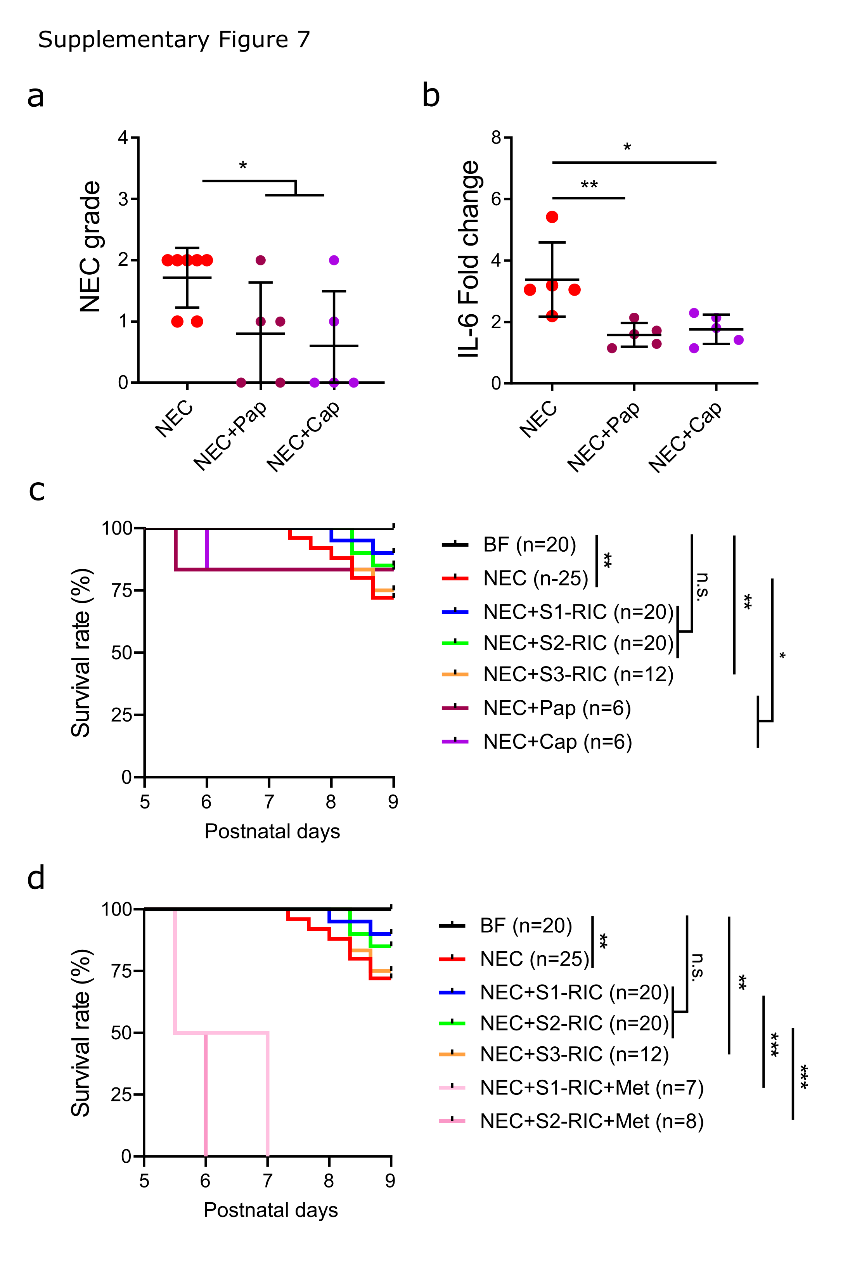


**Supplementary Figure 7.** Improved intestinal perfusion is sufficient to improve the outcome of NEC. **a** Morphology of the ileum was assessed using hematoxylin and eosin staining and was compared between NEC pups (n=7) and NEC pups receiving nonspecific vasodilators, papaverine (n=5) or captopril (n=5). Both papaverine and captopril improved the intestinal damage of NEC. Histological slides were graded by 3 investigators blinded to treatment allocation based on the NEC histopathological scoring system. Mice with histological grade ≥ 2 were considered to have NEC. **b** mRNA expression of proinflammatory marker, *IL-6*, was measured and compared between the listed groups (n=5 per group). Both papaverine and captopril reduced intestinal inflammation in NEC. Data in **a** and **b** were compared using two-sided one-way ANOVA with post hoc Turkey test (*p<0.05; **p<0.01, ***p<0.001) and data are presented as mean ± SEM. **c** Both papaverine and captopril enhanced survival of NEC pups up to sacrifice on P9. **d** Administration of the intestinal vasoconstrictor, methoxamine, abolished the beneficial effect of Stage 1 or 2 RIC in prolonging survival. Survival curves were compared using the logrank test (*p<0.05; **p<0.01). Source data are provided as a Source Data file.


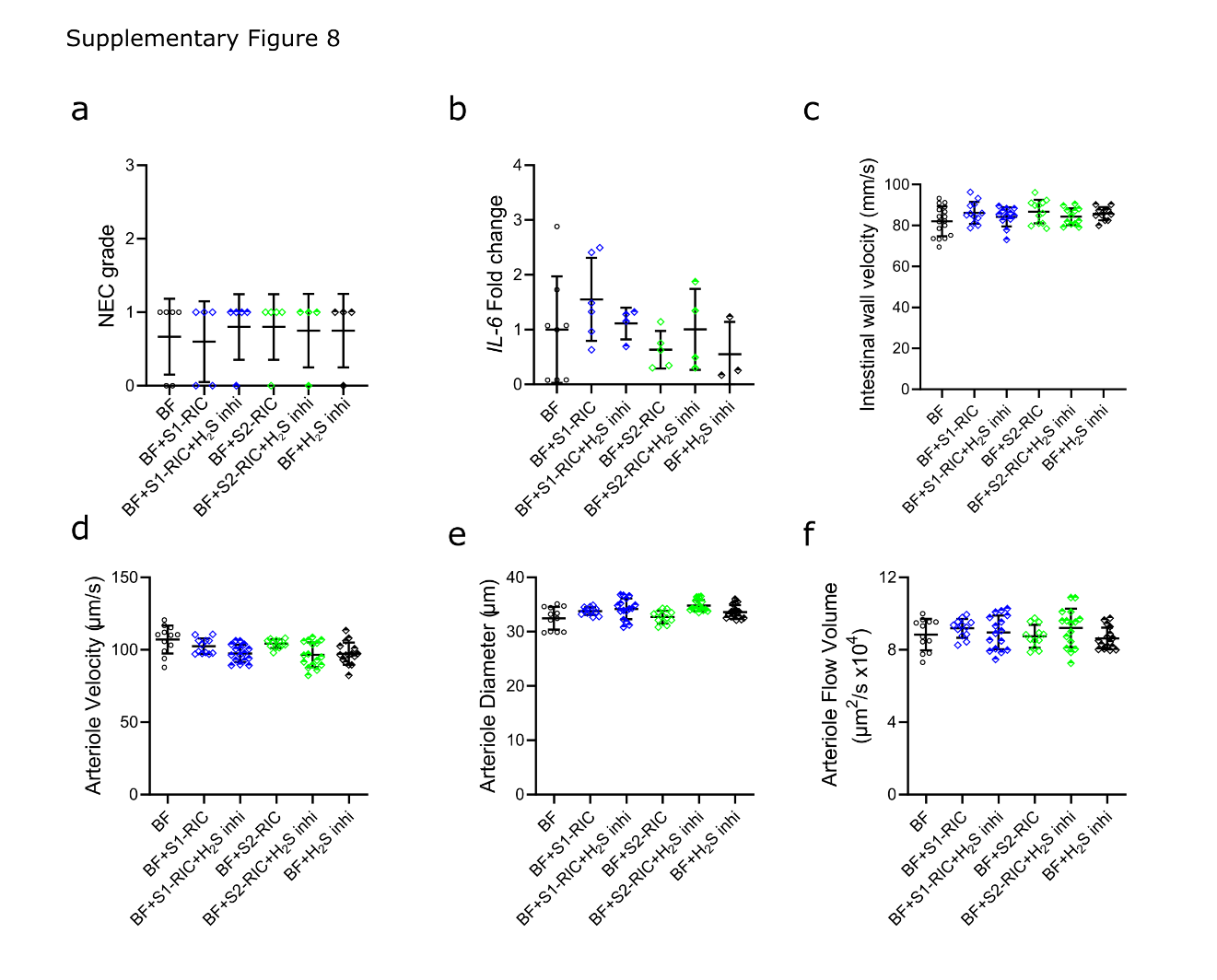


**Supplementary Figure 8.** Stage 1 or 2 RIC in breastfed control pups did not alter intestinal morphology, inflammation, and intestinal microcirculation. **a** Histological scoring of the ileum using hematoxylin and eosin staining and **b** mRNA expression of *IL-6* showed no effect on intestinal injury and inflammation in breastfed control pups receiving Stage 1 or 2 RIC, or in breastfed control pups receiving Stage 1 or 2 RIC and treatment with H_2_S-synthesizing enzyme inhibitors (In **a,** BF: n=6, BF+Stage 1 or 2 RIC: n=5; BF+Stage 1 or 2 RIC+H_2_S inhibitors: n=4. In **b,** BF: n=8; BF+Stage 1 RIC: n=6; BF+Stage 2 RIC: n=5; BF+Stage 1 or 2 RIC+H_2_S inhibitors: n=4). **c** Assessment of intestinal wall velocity (mm/s), **d** submucosal arteriole velocity (µm/s), **e** diameter (µm), and **f** flow volume [(μm)3/s] using TPLSM revealed no effect on intestinal perfusion in breastfed control pups receiving Stage 1 or 2 RIC, or in breastfed control pups receiving Stage 1 or 2 RIC and treatment with H_2_S-synthesizing enzyme inhibitors (n=4 per group, with a minimum of 2 readings obtained per group). Data was compared using two-sided one-way ANOVA with post hoc test, and are presented as mean ± SEM. Source data are provided as a Source Data file.


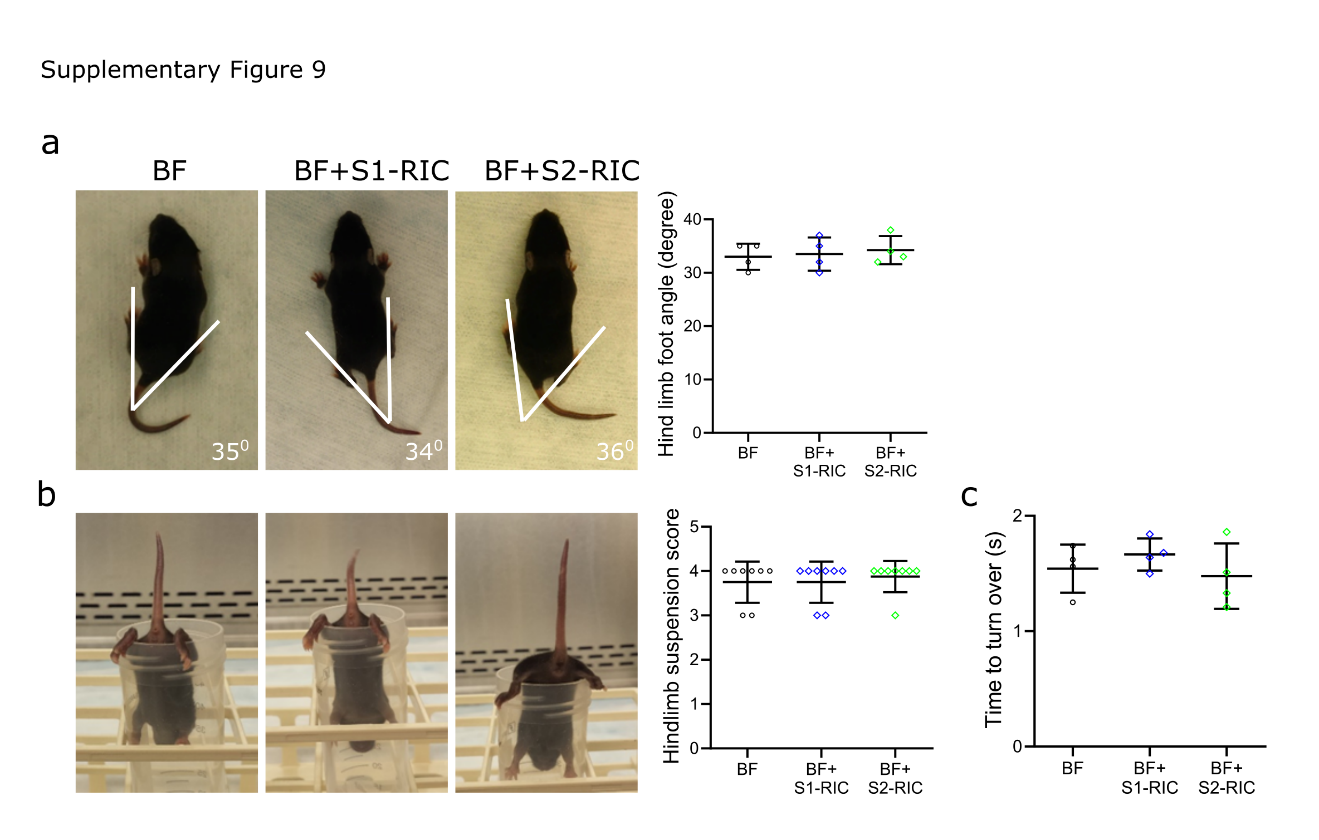


**Supplementary Figure 9.** RIC is a safe maneuver and does not produce any deficits in motor function. **a** The hind limb foot angle test was performed by measuring the foot angle using a line drawn from the mid-heel through the middle (longest) digit. Stage 1 or 2 RIC did not alter the hindlimb foot angle in breastfed control pups (n=4 per group). **b** The hind limb suspension test was performed to evaluate right/left hind limb strength and neuromuscular function. Stage 1 or 2 RIC did not alter the hind limb suspension score in breastfed control pups (n=8 per group). **c** The surface righting test was performed to assess the motor ability of pups to turn over onto their feet from the supine position. Stage 1 or 2 RIC did not alter the time to turn over in breastfed control pups (n=4 per group). Data was compared using two-sided one-way ANOVA with post hoc Turkey test, and are presented as mean ± SEM. Source data are provided as a Source Data file.
