# Supplementary material for: Remote ischemic conditioning counteracts the intestinal damage of necrotizing enterocolitis by improving intestinal microcirculation
Source: Nat Commun. 2020 Oct 2;11:4950. doi: 10.1038/s41467-020-18750-9 (PMC7532542; doi:10.1038/s41467-020-18750-9)
Supplement: Supplementary file 3 — Description of Additional Supplementary Files [file 41467_2020_18750_MOESM3_ESM.pdf]

Title: Supplementary Video 1.
Description: Assessment of intestinal perfusion at P9 in breastfed control pups using Doppler ultrasound.

Title: Supplementary Video 2.
Description: Assessment of intestinal perfusion at P9 in NEC pups using Doppler ultrasound revealed reduced perfusion following NEC induction, compared to breastfed control.

Title: Supplementary Video 3.
Description: Conditioning with Stage 1 RIC during experimental NEC revealed remarkable improvement in intestinal wall perfusion, assessed by Doppler ultrasound.

Title: Supplementary Video 4.
Description: Conditioning with Stage 2 RIC during experimental NEC revealed remarkable improvement in intestinal wall perfusion, assessed by Doppler ultrasound.

Title: Supplementary Video 5.
Description: Microcirculatory perfusion in the submucosa of the intestine at P9 in $Rosa^{GFP}$ breastfed control pups, recorded using TPLSM. Recording depicts arterioles in the submucosa.

Title: Supplementary Video 6.
Description: Microcirculatory perfusion in the submucosa of the intestine at P9 in $Rosa^{GFP}$ NEC pups, recorded using TPLSM. NEC induction resulted in decreased diameter and blood velocity of submucosal arterioles.

Title: Supplementary Video 7.
Description: Microcirculatory perfusion in the submucosa of the intestine at P9 in $Rosa^{GFP}$ NEC pups conditioned with Stage 1 RIC, recorded using TPLSM. Conditioning with Stage 1 RIC resulted in remarkable preservation of the diameter and blood velocity of submucosal arterioles.

Title: Supplementary Video 8.
Description: Microcirculatory perfusion in the submucosa of the intestine at P9 in $Rosa^{GFP}$ NEC pups conditioned with Stage 2 RIC, recorded using TPLSM. Conditioning with Stage 2 RIC resulted in remarkable preservation of the diameter and blood velocity of submucosal arterioles.

Title: Supplementary Video 9.
Description: Necrosis in the intestinal epithelium in $Rosa^{mT/mG/+}$;Tie2-Cre breastfed control pups at P9 detected by Sytox Green staining.

Title: Supplementary Video 10.
Description: Necrosis in the intestinal epithelium in $Rosa^{mT/mG/+}$;Tie2-Cre NEC pups at

P9 detected by Sytox Green staining. Necrosis of enterocytes was increased following NEC induction, especially at the villi tip.

Title: Supplementary Video 11.
Description: Necrosis in the intestinal epithelium in Rosa$^{mT/mG/+}$;Tie2-Cre NEC pups conditioned with Stage 1 RIC at P9 detected by Sytox Green staining. Following conditioning with Stage 1 RIC, necrosis of enterocytes at the villi tip was remarkably reduced.

Title: Supplementary Video 12.
Description: Necrosis in the intestinal epithelium in Rosa$^{mT/mG/+}$;Tie2-Cre NEC pups conditioned with Stage 2 RIC at P9 detected by Sytox Green staining. Following conditioning with Stage 2 RIC, necrosis of enterocytes at the villi tip was remarkably reduced.

Title: Supplementary Video 13.
Description: Assessment of intestinal perfusion at P9 in NEC pups receiving Stage 1 RIC and treatment with H_2S-synthesizing enzyme inhibitors using Doppler ultrasound. The RIC-mediated preservation of intestinal perfusion during NEC was abolished following inhibition of H_2S synthesis.

Title: Supplementary Video 14.
Description: Assessment of intestinal perfusion at P9 in NEC pups receiving Stage 2 RIC and treatment with H_2S-synthesizing enzyme inhibitors using Doppler ultrasound. The RIC-mediated preservation of intestinal perfusion during NEC was abolished following inhibition of H_2S synthesis.

Title: Supplementary Video 15.
Description: TPLSM assessment of microcirculatory perfusion in the submucosa of the intestine at P9 in Rosa^{GFP} NEC pups conditioned with Stage 1 RIC and treated with H_2S-synthesizing enzyme inhibitors. The RIC-mediated enhancement of blood velocity in submucosal arterioles during NEC was abolished following inhibition of H_2S synthesis.

Title: Supplementary Video 16.
Description: TPLSM assessment of microcirculatory perfusion in the submucosa of the intestine at P9 in Rosa^{GFP} NEC pups conditioned with Stage 2 RIC and treated with H_2S-synthesizing enzyme inhibitors. The RIC-mediated enhancement of blood velocity in submucosal arterioles during NEC was abolished following inhibition of H_2S synthesis.

Title: Supplementary Video 17.
Description: Surface righting test of breastfed control pups.

Title: Supplementary Video 18.
Description: Surface righting test of breastfed control pup conditioned with Stage 1 RIC revealed no alterations due to conditioning with RIC.

Title: Supplementary Video 19.
Description: Surface righting test of breastfed control pups conditioned with Stage 2 RIC revealed no alterations due to conditioning with RIC.
