# Supplementary material for: Remote ischemic conditioning counteracts the intestinal damage of necrotizing enterocolitis by improving intestinal microcirculation
Source: Nat Commun. 2020 Oct 2;11:4950. doi: 10.1038/s41467-020-18750-9 (PMC7532542; doi:10.1038/s41467-020-18750-9)
Supplement: Supplementary file 23 — Reporting Summary [file 41467_2020_18750_MOESM23_ESM.pdf]

nature research

Corresponding author(s): Agostino Pierro

Last updated by author(s): 8/31/2020

Reporting Summary

Nature Research wishes to improve the reproducibility of the work that we publish. This form provides structure for consistency and transparency in reporting. For further information on Nature Research policies, see Authors & Referees and the Editorial Policy Checklist .

Statistics

For all statistical analyses, confirm that the following items are present in the figure legend, table legend, main text, or Methods section.

n/a	Confirmed	
☐	☒	The exact sample size (*n*) for each experimental group/condition, given as a discrete number and unit of measurement
☐	☒	A statement on whether measurements were taken from distinct samples or whether the same sample was measured repeatedly
☐	☒	The statistical test(s) used AND whether they are one- or two-sided<br>*Only common tests should be described solely by name; describe more complex techniques in the Methods section.*
☒	☐	A description of all covariates tested
☐	☒	A description of any assumptions or corrections, such as tests of normality and adjustment for multiple comparisons
☐	☒	A full description of the statistical parameters including central tendency (e.g. means) or other basic estimates (e.g. regression coefficient) AND variation (e.g. standard deviation) or associated estimates of uncertainty (e.g. confidence intervals)
☐	☒	For null hypothesis testing, the test statistic (e.g. *F*, *t*, *r*) with confidence intervals, effect sizes, degrees of freedom and *P* value noted<br>*Give P values as exact values whenever suitable.*
☒	☐	For Bayesian analysis, information on the choice of priors and Markov chain Monte Carlo settings
☒	☐	For hierarchical and complex designs, identification of the appropriate level for tests and full reporting of outcomes
☒	☐	Estimates of effect sizes (e.g. Cohen's *d*, Pearson's *r*), indicating how they were calculated

Our web collection on statistics for biologists contains articles on many of the points above.

Software and code

Policy information about availability of computer code

Data collection	ZEN 2.0 lite software was used for collection of intestinal perfusion data in vivo via two photon laser scanning microscopy (TPLSM).
Data analysis	Statistical analysis was performed using GraphPad prism V8. ZEN 2.0 lite software was used for quantification of data obtained by two photon laser scanning microscopy (TPLSM). Immunofluorescent images were quantified using ImageJ.

For manuscripts utilizing custom algorithms or software that are central to the research but not yet described in published literature, software must be made available to editors/reviewers. We strongly encourage code deposition in a community repository (e.g. GitHub). See the Nature Research guidelines for submitting code & software for further information.

Data

Policy information about availability of data

All manuscripts must include a data availability statement. This statement should provide the following information, where applicable:

- Accession codes, unique identifiers, or web links for publicly available datasets
- A list of figures that have associated raw data
- A description of any restrictions on data availability

All datasets associated with this study are available in the main text or the supplementary materials. Source data are provided with this paper as Excel sheets uploaded with the Supplementary Information.

Field-specific reporting

Please select the one below that is the best fit for your research. If you are not sure, read the appropriate sections before making your selection.

Life sciences study design

All studies must disclose on these points even when the disclosure is negative.

Sample size	The relevant samples size relates to the number of mice analyzed. In all experiments individual animals were analyzed. Experiments were designed to include multiple mice per group and to include multiple independent experiments. For each individual experiment, the sample size was not predetermined before initial experiment. Normally 3 to 25 biological repeats were included based on experience or the sample availability.
Data exclusions	No data were excluded from analysis.
Replication	All experiments were independently repeated at least three times. All attempts at replication were successful.
Randomization	All animals were randomly assigned to each of the study groups.<br>Human samples obtained in a retrospective manner and from the Division of Pathology at the Hospital for Sick Children.
Blinding	Assessment of histological scoring, immunofluorescence, Doppler ultrasound, TPLSM, and survival were conducted by investigators blind to the allocation of treatment.

Reporting for specific materials, systems and methods

We require information from authors about some types of materials, experimental systems and methods used in many studies. Here, indicate whether each material, system or method listed is relevant to your study. If you are not sure if a list item applies to your research, read the appropriate section before selecting a response.

Materials & experimental systems

n/a	Involved in the study	
	x	Antibodies
x		Eukaryotic cell lines
x		Palaeontology
	x	Animals and other organisms
	x	Human research participants
x		Clinical data

Methods

n/a	Involved in the study	
x		ChIP-seq
x		Flow cytometry
x		MRI-based neuroimaging

Antibodies

Antibodies used	Polyclonal rabbit anti-CBS antibody (Proteintech, 14787-1-AP) (1:500), Hif1-α (mouse monoclonal, Novus NB100-105) (1:250), CD31 (rabbit polyclonal Abcam ab18364) (1:100), Alexa Fluor-conjugated secondary antibody (Invitrogen, Carlsbad, California, United States) (1:1000), DAPI (Vector Laboratories) (1:1000), pimonidazole (Hypoxyprobe, Vector Laboratories, Burlington, ON)
Validation	polyclonal rabbit anti-CBS antibody:<br>Validation: Manufacturer - https://www.ptglab.com/products/CBS-Antibody-14787-1-AP.htm#validation<br><br>pimonidazole:<br>Validation: Manufacturer - http://site.hypoxyprobe.com/knowledge-center-articles/HP-1-Kit-Insert.pdf<br><br>Hif1-α:<br>Validation: Manufacturer - https://www.novusbio.com/products/hif-1-alpha-antibody-h1alpha67_nb100-105#datasheet<br><br>CD31:<br>Validation: Manufacturer - https://www.abcam.com/cd31-antibody-ab28364.html

Animals and other organisms

Policy information about studies involving animals; ARRIVE guidelines recommended for reporting animal research

Laboratory animals	The study used C57BL/6 mice (male and female), GFP mice (male and female), RosamT/mG/;Tie2-Cre mice (male and female), and eNOS knockout mice (male and female). Mouse pups were separated from corresponding breeding pairs at Postnatal day 5 (P5), and subjected to the NEC protocol from P5 to P9, as described in the manuscript.
Wild animals	The study did not involve wild animals.
Field-collected samples	The study did not involve samples collected from the field.

Ethics oversight	All animal experiments were approved by the Animal Care Committee of the Hospital for Sick Children, Toronto, Ontario (ethics no. 32238).

Note that full information on the approval of the study protocol must also be provided in the manuscript.

Human research participants

Policy information about studies involving human research participants

Population characteristics	All infants were premature and were operated on during the first 7 weeks of life. The ileum was resected during emergency laparotomy for "acute active NEC" (n=5). Age-matched control samples (n=5) were obtained from resected ileum of infants undergoing surgery for less-severe diseases of the intestine (Hirschsprung's disease, meconium ileus).
Recruitment	Samples were obtained from the terminal ileum that was excised during surgery and stored in the Division of Pathology at the Hospital for Sick Children. These samples were obtained in a retrospective manner and with no self-selection bias.
Ethics oversight	Ethical approval for this study was obtained from the Research Ethics Board of the Hospital for Sick Children, Toronto, Canada (protocol #1000056881). Informed consent was obtained from the Legally Authorized Representatives of infants prior to surgery. All methods performed in the study were carried out in accordance with the approved guidelines and regulations. Tissue analysis was done with approval from the Hospital for Sick Children and in accordance with anatomical tissue procurement guidelines.

Note that full information on the approval of the study protocol must also be provided in the manuscript.
